# The traditional Chinese medicine and non-small cell lung cancer: from a gut microbiome perspective

**DOI:** 10.3389/fcimb.2023.1151557

**Published:** 2023-04-25

**Authors:** Xuelin Wang, Liming Hou, Meng Cui, Junnan Liu, Mengzhou Wang, Jianwu Xie

**Affiliations:** ^1^ School of Food Science and Engineering (School of Biological and Pharmaceutical Sciences), Shaanxi University of Science & Technology, Xi an, China; ^2^ Department of Geriatrics, Xijing Hospital, Fourth Military Medical University, Xi an, China

**Keywords:** NSCLC, gut microbiome, lung-gut axis, TCM, Chinese herbal compounds

## Abstract

Non-small cell lung cancer (NSCLC) is one of the most serious diseases affecting human health today, and current research is focusing on gut flora. There is a correlation between intestinal flora imbalance and lung cancer, but the specific mechanism is not clear. Based on the “lung and large intestine being interior-exteriorly related” and the “lung-intestinal axis” theory. Here, based on the theoretical comparisons of Chinese and western medicine, we summarized the regulation of intestinal flora in NSCLC by active ingredients of traditional Chinese medicine and Chinese herbal compounds and their intervention effects, which is conducive to providing new strategies and ideas for clinical prevention and treatment of NSCLC.

## 1 Introduction

In the womb, the fetus begins to develop its gut microbiota, and an estimated 40 trillion microorganisms are considered to reside on and in the human body. The digestive tract, often known as the “gut microbiome (GM),” contains the most microbial species variety. (Nagasaka et al., 2020). Although its primary function has been thought to be to protect against pathogen overgrowth in the gut, the gut microbiome appears to play a critical role in the maturation and ongoing education of the host immune response and likely has significant effects in many conditions not typically considered infectious diseases (Fulde and Hornef, 2014; Kamada et al., 2013). Intestinal microbiome imbalances, also known as ecological imbalances, have been linked to a variety of illnesses, including cancer, in recent years. Given that the gut microbiome is constantly exposed to a wide spectrum of potential pathogens, it is not surprising that it is vital to the host immune response.

According to the latest data from China’s National Cancer Center in 2022, lung cancer is the second most common cancer in humans worldwide, with the highest incidence of morbidity and mortality. NSCLC accounts for more than 80% of all lung cancers, and it is one of the most challenging to treat with a poor response to immune checkpoints (ICIs) in most patients. Interestingly, emerging evidence has suggested that microbiota may also play vital roles in lung cancers at multiple levels (Vernocchi et al., 2020).

Additionally, there is mounting evidence linking the GM and its metabolome to the response to ICI treatment in NSCLC (Jin et al., 2019; Botticelli et al., 2020; Hakozaki et al., 2020). In fact, the metabolites of the microbes as well as their cells contribute to the stimulation of the immune response. Their interactions stimulate and trigger an immunological response, helping the host immune system combat cancer. The relationship between the gut microbiota and non-small cell lung cancer will therefore be the main topic of this essay.

## 2 Close relationship: GM and NSCLC

### 2.1 The overview of GM

**Figure 1:**
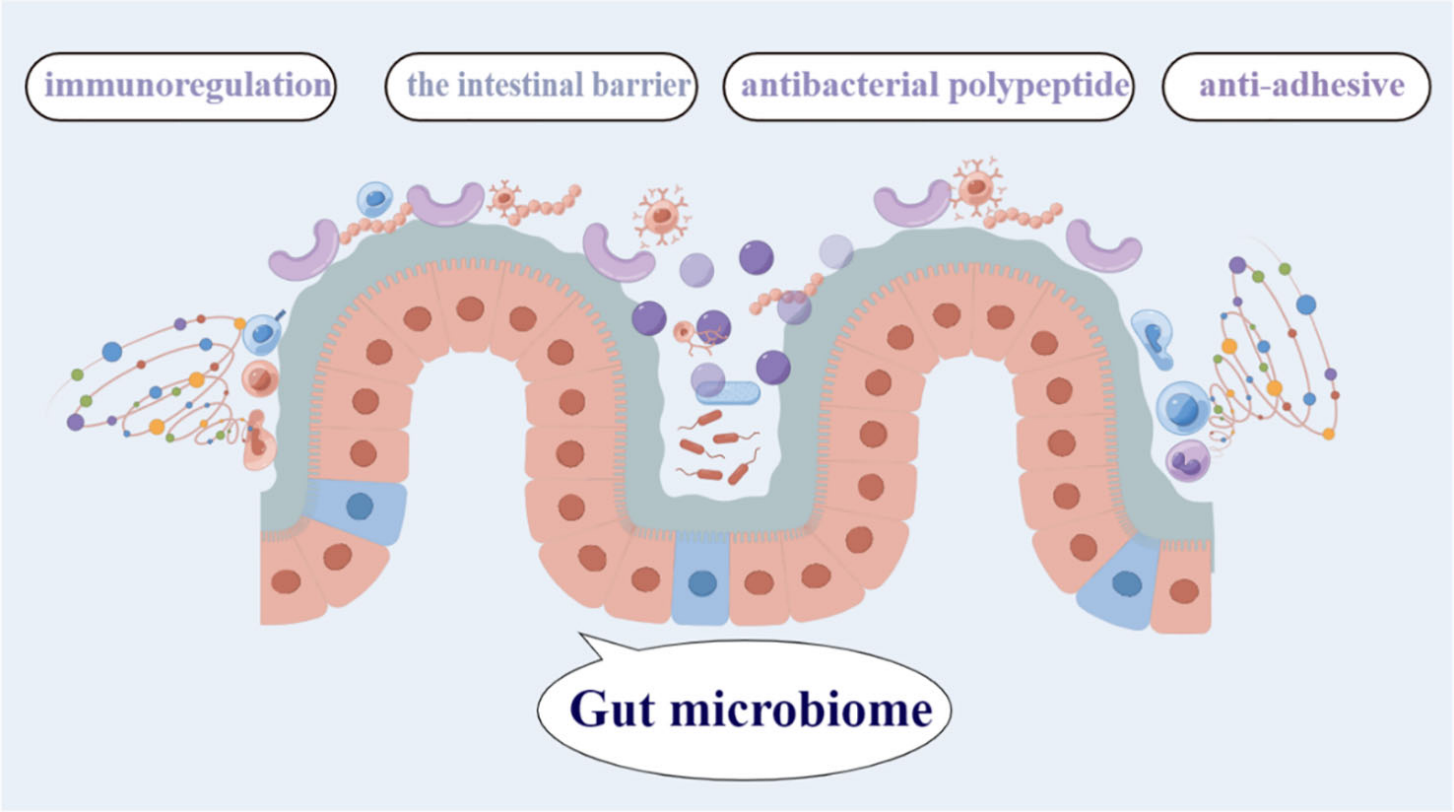
The gut microbiome contains the greatest diversity of microbial species, and plays a critical role in the immunoregulation, antibacterial polypeptide, anti-adhesive,the intestinal barrier,and so on.

A wide variety of microorganisms, including bacteria, fungus, archae, and viruses, live in the complex, dynamic, and geographically heterogeneous ecosystem known as the human GM (Chen et al., 2021b). The total genetic repertory of all gut microbes is an order of magnitude greater than the genetic repertoire of the human genome, and also encodes many more unique genes than the host genome, and they generate more than 1000 metabolites (Fan and Pedersen, 2021). It is also referred to as the “essential organ” of the human body (Ding et al., 2019). The GM is the body’s greatest micro-ecosystem and works in symbiosis with the host to sustain regular physiological functions in a state of dynamic balance (**Figure 1**). Human host receives a number of crucial services from the gut microbiota attests to its significance, including the conversion of indigestible dietary components, the creation of vital vitamins, the elimination of harmful substances, the defeat of infections, the augmentation of the intestinal barrier, and the stimulation and control of the immune system (Heintz-Buschart and Wilmes, 2018), these are necessary to support normal tissue and organ function. It is generally known that the gut microbiota directly affects both health and disease status. The microbiome has a significant impact on host physiology due to its broad metabolic and synthetic capabilities and its intricate interactions with the formation and control of the host immune system (Walker et al., 2021). Despite the symbiotic nature of the intestinal host-microbial relationship, the close association of an abundant bacterial community with intestinal tissues poses immense health challenges (Hooper et al., 2012).

### 2.2 The composition of GM

The development of the technique to sequence the bacterial 16S ribosomal RNA gene allowed overall taxonomic assessment of the gut microbiome, and this has dramatically increased our knowledge of the broad variations in microbial composition (Weersma et al., 2020). The human gut microbiota consists of several types of microbes, including bacteria, archaea, eukarya, viruses, and parasites. More than 1000 species of bacteria have been identified in the human gut, although a person on average only carries 160 species (Berding et al., 2021). The gut microenvironment mainly favors the growth of bacteria from seven predominant divisions (Firmicutes, Bacteroidetes, Actinobacteria, Fusobacteria, Proteobacteria, Verrucomicrobia, and Cyanobacteria). Among these seven divisions, the Bacteroidetes and Firmicutes constitute more than 90% of the total population (Adak and Khan, 2019). Studies indicate links between dysbiosis or disturbance in the microbiome and diseases that not only affect the gut but also organs like the lung, thyroid, brain, cardiac, immune system, etc. (Tang et al., 2019; Hufnagl et al., 2020; Knezevic et al., 2020; Rutsch et al., 2020; Wang et al., 2022a). The crosstalk between the gut microbiome and distal organs is being increasingly recognized, and host-microbiome interactions are being delineated piece by piece. Gut microbes and their associated metabolites are thought to cause and modulate lung cancer development, albeit influenced by the host genetic make-up and environment. Non-targeted metabolomics approach based on LC-MS can successfully distinguish lung cancer patients from healthy individuals. Also, the microbial diversity in lung cancer patients is significantly higher than that of normal individuals (Zhao et al., 2021).

### 2.3 The role of GM in NSCLC (Lung-gut axis)

**Figure 2:**
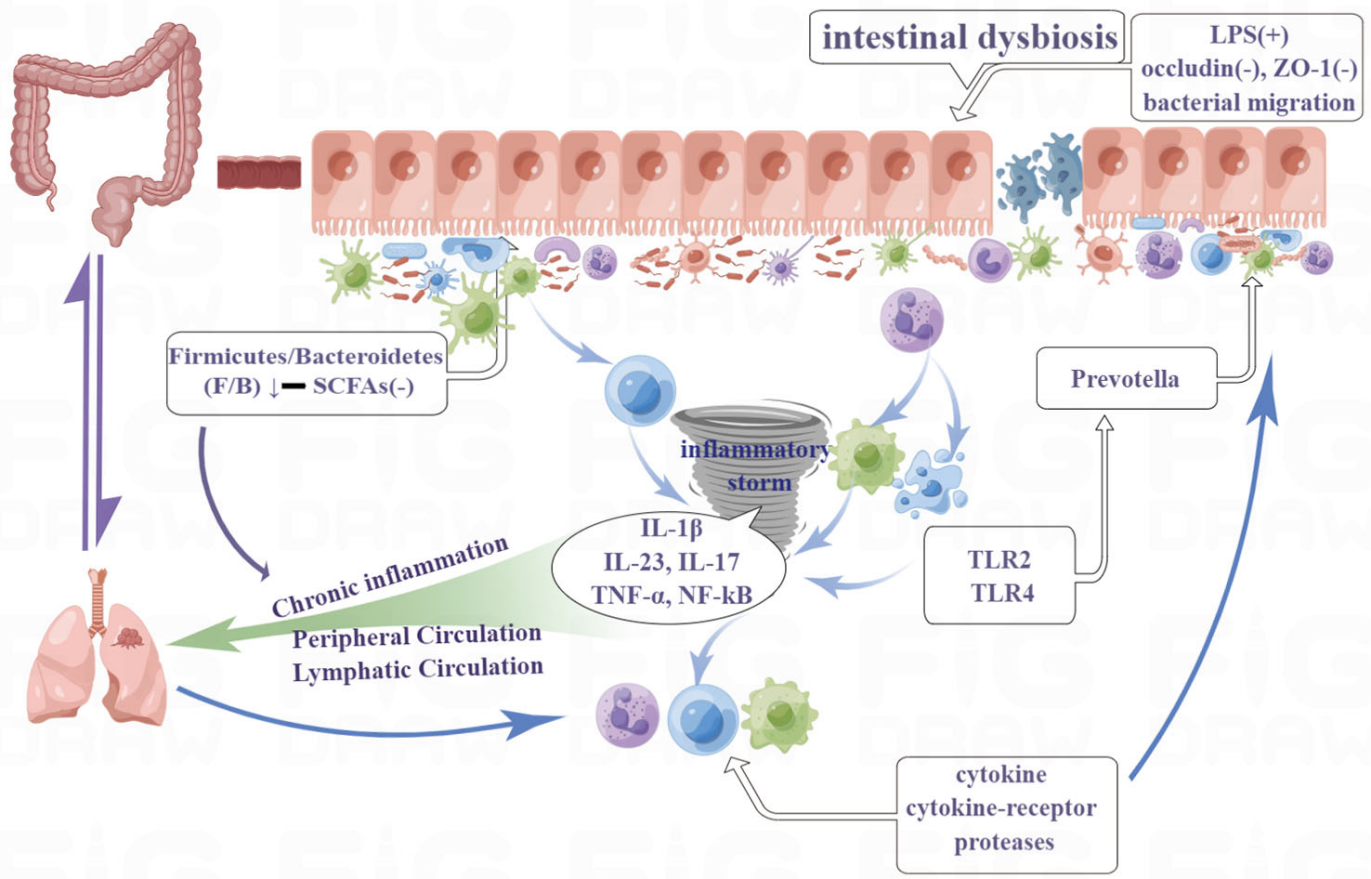
The lungs and large intestine can work together to modulate immunity and inflammation through the lung-gut axis, in which the movement of the gut microbiota and metabolites is the most important communication mechanism. Moreover, they are indirectly connected via the circulatory and lymphatic systems.

Chinese medicine believes that “the lung and large intestine are interior-exteriorly related” (Ni et al., 2022). The hypothesis of the lung-gut axis, which modern medicine has advanced, corresponds to the Chinese medical theory of the “lung and large intestine being interior-exteriorly related.” The lungs and large intestine can work together to modulate immunity and inflammation through the lung-gut axis, in which the movement of the gut microbiota and metabolites is the most important communication mechanism (**Figure 2**). The lungs and the gut both develop from the same embryo. The gut and the lungs, like all other organs included in the MIS compartments, are mucosally similar, promoting comparable dynamics in the interactions between the immune system and their microbiota. Moreover, they are indirectly connected *via* the circulatory and lymphatic systems (Pizzo et al., 2022). The lungs do indeed have a specific microbiota. The predominant bacterial phyla in the lungs of healthy subjects are the same as those in the gut. These are mainly Firmicutes (Staphylococcus, La-ctobacillus, and Streptococcus) and Bacteroidetes, followed by Proteobacteria and Actinobacteria (Georgiou et al., 2021). While lung cancer patients present higher levels of Bacteroidetes, Fusobacteria, Cyanobacteria, Spirochaetes, and Lentisphaerae, and lower levels of Bacteroidetes, Firmicutes, and Verrucomicrobia in their lung and gut microbiota (Corrêa et al., 2022).

Although the gastrointestinal and respiratory systems are separated by physical distance, they share a common embryonic origin and exhibit a striking structural similarity, suggesting the possibility of multimodal interaction between these two tracts. As a result, the gut-lung axis, a new and distinct interaction between the respiratory and gastrointestinal tracts, has been created. According to reports, this two-way regulation of the gut-lung axis organs is accomplished through microbial and immunological processes. A growing body of research suggests that the microbiome is crucial in inflammatory pulmonary disorders such as acute lung injury (ALI) and acute respiratory distress syndrome. Tang et al. discovered that the transition from ulcerative colitis (UC) to colorectal cancer (CRC) significantly altered not only the composition of the gut microbiota and metabolites associated with inflammation but also the lung tissues, which demonstrated that gastrointestinal illnesses can result in pulmonary illnesses (Tang et al., 2022). Yoon et al. found that the composition of the gut microbiota has a significant impact on BLM-induced wasting and death, suggesting a role for the lung-gut axis in lung injury. They also found that the presence of specific gut commensal microbes may be a risk factor for having more severe inflammatory lung diseases (Yoon et al., 2022).

#### 2.3.1 GM regulated inflammation and immune system

It’s vital to keep in mind that inflammation can have two opposing impacts on tumors: whereas local inflammation limited to the tumor microenvironment can reduce the tumor, chronic, broad inflammation generally promotes tumor growth. According to preclinical research in mouse models (Sánchez-Alcoholado et al., 2020), GM-mediated colorectal cancer (CRC) and inflammation have a high correlation. Guo et al. revealed that *Ganoderma lucidum* (GLP) decreased colitis and tumorigenesis. The potential explanation is that GLP ameliorated microbiota dysbiosis, increased short-chain fatty acid production, profoundly improved gut barrier function as evidenced by increased numbers of goblet cells, MUC2 secretion, and tight junction protein expressions. Simultaneously, GLP treatment inhibited macrophage infiltration and downregulated IL-1β, iNOS, and COX-2 expressions (Guo et al., 2021a).

The gut microbiome plays a key role in intestinal permeability and immune regulation. The gut microbiome regulates immune cell populations in part through short-chain fatty acids, which can restore colonic regulatory T cell populations in germ-free mice and signalling *via* Toll-like receptors (TLRs) among other innate and adaptive immune pathways (Leigh and Morris, 2020). Peng et al. recruited 74 patients with advanced-stage gastrointestinal (GI) cancer receiving anti-PD-1/PD-L1 treatment and collected their fecal samples prior to and during immunotherapy, along with clinical evaluations. They revealed an elevation of the *Prevotella/Bacteroides* ratio in patients, with a preferred response to antiPD-1/PD-L1 treatment and a particular subgroup of responders harboring a significantly higher abundance of *Prevotella, Ruminococcaceae*, and *Lachnospiraceae* (Peng et al., 2020). Huang et al. found Ginseng polysaccharides (GPs) increased the antitumour response to αPD-1 monoclonal antibody (mAb) by increasing the microbial metabolites valeric acid and decreasing L-kynurenine, as well as the ratio of kynurenine/tryptophan, which contributed to the suppression of regulatory T cells and induction of Teff cells after combination treatment. And the microbial analysis indicated that the abundance of *Parabacteroides distasonis and Bacteroides vulgatus* was higher in responders to anti-PD-1 blockade than in non-responders in the clinic(Huang et al., 2022). And a clinical study of 37 patients with advanced NSCLC in China reveal strong correlation between gut microbiome diversity and the responses to anti-PD-1 immunotherapy. Patients with a favorable gut microbiome exhibit enhanced memory T cell and natural killer cell signatures in the periphery (Jin et al., 2019).

#### 2.3.2 Metabolites of GM in NSCLC

The short-chain fatty acids (SCFAs), which are the major metabolic products of the GM from dietary fiber (especially in the case of a high-fiber diet), are key mediators of the host–microbiome interaction and perform countless functions with localized and systemic effects. Main SCFAs with total intestinal concentrations exceeding 100 mM include propionate, acetate, and butyrate (Mirzaei et al., 2021). The basic function of these fatty acids is to provide energy. SCFAs also act as signaling molecules by mediating metabolic processes and immune responses, and various studies have proven their impressive anti-inflammatory action and antitumor potential. One study showed that sodium butyrate affects proliferation and migration of A549 cells by activating the TNF receptor-associated factor 6 (TRAF6)-thioredoxin-interacting protein (TXNIP) pathway, suggesting that sodium butyrate has an effective therapeutic effect on lung adenocarcinoma. (Xiao et al., 2020). It was also found that sodium butyrate and docetaxel alone, respectively, inhibited proliferation and promoted apoptosis in A549 cells *in vitro* and *in vivo*. Furthermore, the combined therapy decreased protein expression of Ki-67, CDK1, CDK2, Cyclin D1, Bcl-2, and Survivin and increased protein expression of Cyclin A, p21, Bax and cleaved-Caspase 3 (Chen et al., 2020b). Chen et al. reported that propionate and butyrate produced by gut microbiota after probiotic supplementation can attenuate the lung metastasis of melanoma cells in mice (Chen et al., 2021a). Sodium propionate (SP) inhibited lung cancer cell proliferation by inducing cell apoptosis and cell cycle arrest, especially in the G2/M phase (Kim et al., 2019).

#### 2.3.3 GM involved in the treatment of NSCLC

Unusual bacterial clusters were discovered in the NSCLC patients in an observational investigation with exploratory GM analysis, and the individuals who did not have cachexia were enriched with healthy bacteria at the genus level, including Eubacterium, Anaerostipes, and Blautia (Hakozaki et al., 2022). Akkermansia bacteria, specifically Akkermansia mucinifla, is now being found in relation to supporting therapy and markers for an immunotherapy response in cancer patients. It has been shown that this bacterium improves response to treatment in NSCLC patients receiving immune checkpoint inhibitors (ICIs). In the 47 NSCLC patients studied by Grenda et al., patients with disease stabilization and partial immunotherapy responses had a higher percentage of Akkermansiaceae than patients with cancer progression. Additionally, they discovered that patients with squamous cell carcinoma had more Akkermansiaceae than those with adenocarcinoma. As a result, they suggested that Akkermansiaceae may serve as a supportive marker for NSCLC patients’ immunotherapy responses (Grenda et al., 2022). An animal research indicated that Akkermansia muciniphila (Akk) combining with cisplatin (CDDP) slowed down the growth of tumor volume and improved the changes in tumor pathomorphology and was related to those pathways, including the cytokine-cytokine receptor interaction, Th17 cell differentiation, FOXO, JAK-STAT, and PI3K-Akt signaling pathways (Chen et al., 2020c).

Cancer immunotherapy has become highly successful against an array of distinct hematological and solid metastatic malignancies. Immune checkpoint inhibitors (ICIs) targeting the programmed death-1/programmed death-ligand 1 (PD-1/PD-L1) axis induce sustained clinical responses in a measure of cancer patients. Routy et al. found that primary resistance to ICIs can be attributed to abnormal gut microbiome composition (Routy et al., 2018). Their results prove that Fecal microbiota transplantation (FMT) from cancer patients who responded to ICIs into germ-free or antibiotic-treated mice ameliorated the antitumor effects of PD-1 blockade, whereas FMT from nonresponding patients failed to do so. Drug regimens for many tumors also relate to intestinal microecology, such as one of the most popular chemotherapy drugs, paclitaxel (PTX), which was used to treat a variety of tumor types but whoes debilitating side effects included gastrointestinal and behavioral disorders, restricting its use while a 30-day sodium butyrate (BuNa) pre-treatment repaired the altered gut barrier integrity and microbiota composition caused by the PTX. These findings suggest that dietary supplementation with this secure postbiotic may be taken into account for treating PTX-induced central side effects when treating cancer (Cristiano et al., 2022). In addition, probiotic use was linked to better clinical outcomes in patients with advanced or recurrent NSCLC who received anti-PD-1 monotherapy, according to a multicenter retrospective analysis (Takada et al., 2021), which indicates that probiotics may be an superior option for NSCLC patients who receive ICIs.

## 3 Traditional Chinese medicines and GM

### 3.1 Active components of TCMs

#### 3.1.1 Polysaccharide

Drug therapy using natural substances is currently considered a promising future alternative to traditional medicine. As an important class of biologically active natural products, polysaccharides from TCM play an important role in the field of medicine, including gut microbiome regulation (**Table 1**), immune regulation, as well as anti-tumor, anti-oxidation, etc. (Yu et al., 2018). Luo et al., who demonstrated that *Polygonatum sibiricum* polysaccharides-1 (PSP-1) reconstructed the gut microbiota composition, including reducing the relative abundance of Helicobacter, and increasing Akkermansia muciniphila, and revealed that PSP-1 may improve the inflammatory environment and reduce Amyloid-β (Aβ) deposition in the intestine of 5xFAD mice by acting on the bacteria (Luo et al., 2022). Sun et al. found that a water-insoluble polysaccharide (WIP) isolated and identified from the *Poria cocos* mushroom significantly enhanced the butyrate-producing bacteria Lachnospiracea and Clostridium. It was also demonstrated that WIP treatment increased butyrate-producing in the gut, maintained intestinal integrity, and reducted of endoxemia, and activated the intestinal PPAR-γ pathway (Sun et al., 2019). Some scholars have carried out related research on *Astragalus* polysaccharide (APS). Zhong et al. indicated that *Astragalus* mongholicus polysaccharides (mAPS) significantly reduced the *Firmicutes* to *Bacteroidetes* (F/B) ratio and increased the abundance of *Proteobacteria* and *Episilonbacteria.* And mAPS significantly decreased the expression of colonic G-protein-coupled receptors (GPR) 41 and 43, but it had little effect on the profile of fecal short-chain fatty acids (SCFAs) (Zhong et al., 2022). Additionally, Liu et al. demonstrated that APS significantly regulated gut microbial dysbiosis while also recovering the abnormality of fecal metabolism, including glycolysis/gluconeogenesis metabolism and pyruvate metabolism (Liu et al., 2022). Ying et al. aimed to evaluate the protective effect of Cordyceps sinensis polysaccharides (CSP). They found that CSP could increase the abundance of probiotics and decrease pathogenic bacteria. It reduced the side effects of cyclophosphamide (Cy) on intestinal mucosal immunity and gut microbiota (Ying et al., 2020). Turmeric polysaccharides (TPS) were found to increase the abundance of probiotics, such as Lactobacillus and Clostridium-UCG-014, and exert their gut barrier functions through the activation of the aryl hydrocarbon receptor (AhR) to upregulate epithelial tight junction proteins (Yang et al., 2021). Jing et al. used *Codonopsis pilosula* polysaccharides to treat colitis in model mice with Dextran Sulfate Sodium (DSS)-Induced, and they found that this medicine could stimulate the growth of important probiotics, inhibit the growth of pathogenic bacteria, and enhance the production of short-chain fatty acids (Jing et al., 2018).

**Table 1:** The polysaccharides from TCM regulated gut microbiome.

Bioactive ingredients	Detection method	Gut microbiota	Metabolites	mechanism	Ref.
*Polygonatum sibiricum* polysaccharides (PSPs)	16S rRNA gene sequencing.	*Helicobacter*↓, *Akkermansia muciniphila*↑.	Aβ↓, occludin↑, ZO-1↑, TNFα↓, IL-6↓.	Reduces intestinal permeability.	(Luo et al., 2022)
*Poria cocos* mushroom polysaccharides	16S rRNA gene sequencing.	*Lachnospiracea*↑, *Clostridium*↑.	Butyrate↑, TNFα↓, occludin↑, ZO-1↑, LPS↓, PPAR-γ↑.	Maintains intestinal integrity and reduction of endoxemia.	(Sun et al., 2019)
Astragalus mongholicus polysaccharides(mAPS)	16S rRNA gene sequencing.	Proteobacteria↑, Epsilonbacteriathe↑, Bacteroidetes (F/B) ratio↓.	Occludin↑, ZO-1↑, LPS↓, TLR4↓, P-NF-κB↓, NLRP3↓, GRP43↓, GPR41↓.	Modulating the gut microbiota and SCFA-GPR signaling pathways.	(Zhong et al., 2022)
Astragalus polysaccharides (APS)	16S rRNA gene sequencing.	Blautia↑, Lactobacillus↓.	acetate↓, butyrate↓, propionate↓.	regulating glycolysis /gluconeogenesis metabolism and pyruvate metabolism.	(Liu et al., 2022)
*Cordyceps sinensis* polysaccharides (CSP)	16S rRNA gene sequencing.	Lactobacillus↑, Bifidobacterium↑, Bacteroides↑, Clostridium↓, Flexispira↓.	acetic acid↑, propionic acid↑, butyric acid↑, valeric acid↑, total SCFAs↑.	Recover Cy-induced intestinal mucosal immunosuppression	(Ying et al., 2020)
turmeric polysaccharides (TPS)	16S rRNA gene sequencing.	Clostridia-UCG-014↑, Lactobacillus↑, Akkermansia↑, Bacteroides↑, Firmicutes↓	occludin↑, ZO-1↑, acetic acid↑, butyric acid↑, propanoic acid↑, valeric acid↑, total acid↑, I3AM↓, tryptophan↓, IAA↑, IAAId↑.	modulating the gut microbiota, improving microbial metabolites and gut barrier function.	(Yang et al., 2021)
*Codonopsis pilosula* polysaccharides	16S rRNA gene sequencing.	*Bifidobacterium spp.*↑, *Lactobacillus spp.*↑, *Akkermansia spp.*↑, *Desulfovibrio spp.*↓, *Alistipes spp.*↓, *Helicobacter spp.*↓.	acetic acid↑, butyric acid↑, propionic acid↑, isobutyric acid↑, isovaleric acid↑.	stimulated the growth of important probiotics, inhibited the growth of pathogenic bacteria, enhanced the production of short-chain fatty acids,	(Jing et al., 2018)

↑ increase. ↓ decrease.

#### 3.1.2 Saponin

Saponins, glycosides widely distributed in TCM, include a diverse group of compounds characterized by their structure, which contains a steroidal or triterpenoid aglycone and one or more sugar chains (Güçlü-Üstündağ and Mazza, 2007). Which possess a multitude of biological activities such as antitumor activities, antimicrobial activity, antiviral activity, etc. (Kimura et al., 2019; Sharma et al., 2021). In recent years, it has been found that the components of saponins from TCM play a role in disease treatment by regulating intestinal flora (**Table 2**). Akebia saponin D (ASD) has been shown to treat hyperlipidemic rats induced by a high-fat diet by regulating the intestinal microbiota, and it could partially recover both metabolism dysfunction and the intestinal environment through several metabolic pathways and modulation of the microbial community (Zhou et al., 2019). Guo et al. demonstrated that ginsenoside Rg1 possessed a neuroprotective effect on tree shrew model for Alzheimer’s disease, and may have a close association with the microbiota of the large intestine by significantly reducing the abundance of Bacteroidetes (Guo et al., 2021b). Alike, Chen et al. found that ginsenoside Rg1 could mitigate morphine dependence *via* regulation of gut microbiota and inhibit gut microbiota-derived tryptophan metabolism and reduce serotonin (Chen et al., 2022a). *Astragalus* has the effects of anti-tumor, lowering blood pressure, lowering blood sugar, and improving human immunity. Gong et al. found in animal experiments that *Astragaloside* IV plays a hypoglycemic role by regulating intestinal flora and AMPK/SIRT1 and PI3K/AKT pathways (Gong et al., 2021). On the other hand, intestinal microorganisms can also affect the metabolism of saponins *in vivo* (Dong et al., 2017; Zhang et al., 2018).

**Table 2:** The saponins from TCM regulated gut microbiome.

Bioactive ingredients	Detection method	Gut microbiota	Metabolites	mechanism	Ref.
akebia saponin D (ASD)	16S rRNA gene sequencing.	Norank_f_Bacteroidales_S24-7 gro up↑, Lachnospiraceae_NK4A136_ group↑, Prevotella_9↑, Desulfovibri o↑, Ruminococcus_1↑, *Eubacteriu m_coprostanoligenes*_group↑, Lactobacillus↓, Blautia↓.	1-phenylethylamine↑, deoxycholic acid↑, L-glutamate↓, *cis*-9-palmitoleic acid↓.	The correlation between fecal metabolites and microbiota.	(Zhou et al., 2019)
Ginsenosides Re	16S rRNA gene sequencing.	Prevotella↑, Lactobacillus↓, Bacteroides↓.	Glycosidase activities↑.	Ginsenoside Re and gut microbiota regulated each other.	(Zhang et al., 2018)
Ginsenoside Rb1	16S rRNA gene sequencing.	Blautia↓, Allobaculum↓, Turicibacter↑.	Phosphatidylcholine (PC)↑.	Regulated intestinal flora to increase their PC content.	(Lianqun et al., 2021)
Ginsenoside Rg1	16S rRNA gene sequencing.	Lactobacillus salivarius↑, Firmicutes/Bacteroides ratio↑, Bacteroides↓.	Bcl-2↑, Bax↓.	Improved the microbiota imbalance of the large intestine.	(Guo et al., 2021b)
Ginsenoside Rg1	16S rRNA gene sequencing.	Bacteroidetes↑, Firmicutes↑, Cyanobacteria↓, Proteobacteria↓.	5-HTR1B↓, 5-HTR2A↓, serotonin↓.	Manipulation of the gut microbial composition and tryptophan metabolite.	(Chen et al., 2022a)
Astragaloside IV	16S rRNA gene sequencing.	Anaerobacter↑, Romboutsia↑, Alkalibacteria↑, Canadidatus stoquefichus↑, Oligobacterium↑, Brautella↑, Erysipelatoclostridum↑, Bacteroides↓, Oscillibacter↓, Parabacteroides↓, Roseburia↓, Muribaculum↓.	butyric acid↑.	Regulating gut microbiota and AMPK/SIRT1 and PI3K/AKT signaling pathways.	(Gong et al., 2021)

↑ increase. ↓ decrease.

#### 3.1.3 Flavonoids

*Smilax china L*., commonly known as “Baqia” is not just a comestible; it was also used as traditional herbal medicine in China. It contains multifarious naturally bioactive compounds, such as flavonoids, polyphenols, and steroidal saponins (**Table 3**). Li et al. investigated the effects of *Smilax* chinensis *L*. flavonoid (SCF) on obesity and changes in gut microbiota. Their results found that SCF modulated the composition of gut microbiota and decreased the production of SCFAs, resulting in reduced energy absorption and subsequent weight loss in the mice (Li et al., 2021). The findings of Wang et al. provide evidence that *Acanthopanax senticosus* total flavonoids (ASTFs) have significant anti-inflammatory properties on LPS-induced intestinal inflammation, preserve the integrity of the intestinal barrier, and regulate gut microbiota homeostasis (Wang X. et al., 2022). Baicalin has a variety of pharmacological effects, including anti-inflammation, anti-infection, anti-apoptosis, anti-allergy, and so on. Some scholars have found that baicalin rebalances the gut microbial composition pattern impaired by ionizing radiation (IR) and alleviates IR-induced apoptosis of the gut microbiota (Wang et al., 2020b). In addition, other scholars have also done research on baicalin; they found that baicalin can improve abnormal metabolism and gut microbiota in high-fat diet (HFD)-induced metabolic syndrome (MetS) in mice (Lin et al., 2022). Glycosides and flavonoids from *P. thomsonii* leaves (PL) alleviated type 2 diabetes in high-fat diet plus streptozotocin-induced mice. This process may be associated with the biological activity that Glycosides and flavonoids from PL could increase intestinal probiotics to improve metabolic disorders caused by diabetes and decrease the level of Clostridium celatum to relieve inflammation (Zhang et al., 2022).

**Table 3:** The flavonoids from TCM regulated gut microbiome.

Bioactive ingredients	Detection method	Gut microbiota	Metabolites	mechanism	Ref.
*Smilax china L.* flavonoid (SCF)	16S rDNA gene sequencing.	unclassified_g_Akkermansia↑, uncultured_bacterium_g_Desulfovibrio↓, uncultured_bacterium_g_ Faecalibaculum↓, unclassified _g_norank_ f_Lachnospiraceae↓, uncalssified_g_Lachnoclostridium↓, uncalssified_g_Lactobacilus↓.	propionic acid (PA) ↓, butyric acid (BA) ↓, isobutyric acid (IBA) ↓, valeric acid (VA) ↓, isovaleric acid (IVA) ↓.	Modulating the composition of gut microbiota and decreased the production of SCFAs.	(Li et al., 2021)
*Acanthopanax senticosus* total flavonoids (ASTFs)	16S rRNA gene sequencing.	Ruminiclostridium-9↑, Bacteroides↓, Alistipes↓, Parabacteroides↓.	Goblet cells (GCs)↑, inflammatory cells↓, the small intestine tissue returned to normal.	Preserving the integrity of the intestinal barrier and regulating gut microbiota homeostasis.	(Wang et al., 2022b)
Baicalin	16S rRNA gene sequencing.	Lactobacillus↑, Prevotellaceae_UCG-001↑, Bacteroides↓, Alistipes↓, Parabacteroides↓, Ruminococcaceae_UCG-014↓.	Caspase3↓. Bcl-xl↓. Bax↓, Caspase8↓, p53↓.	Alleviating ionizing radiation (IR) induced apoptosis of gut microbiota.	(Wang K. et al., 2020)
Baicalin	16S rDNA gene sequencing.	Streptococcus↓, Lactococcusm↓, Ruminococcus↓, Oscillospira↓, Lactobacillus↓, Mucispirillum↓, Bacteroides↓, Odoribacter↓, Butyricimonas↓.	Regulated TCA cycle and Aminoacyl-tRNA biosynthesis.	Regulating metabolites and gut microbiota.	(Lin et al., 2022)
Glycosides and flavonoids from *P. thomsonii* leaves	16S rRNA gene sequencing.	Lactobacillus↑, Clostridium celatum↓, Firmicutes/Bacteroidetes (F/B) ↓.	activated carbohydrate biosynthesis and glycolysis.	Improved metabolic disorders and relieved inflammation.	(Zhang et al., 2022)

↑ increase. ↓ decrease.

#### 3.1.4 Alkaloid

Alkaloid is an important natural organic compound that is one of the important effective components in Chinese herbal medicine. It has a variety of biological activities and pharmacological effects, mainly antibacterial, anti-inflammatory, liver protection, and nervous system effects. It has been confirmed that the components of alkaloids from TCM play a role in disease treatment by regulating gut microbiota (**Table 4**). Zhang et al. confirmed that alkaloids from *Sophora alopecuroides* L. can improve depression in mice; this biological phenomenon is associated with modulating gut microbiota and gut microbiota directly participating in the metabolic process of the host (Zhang et al., 2021a). Berberine, the major active ingredient of the Chinese herb Coptis chinensis (Huang-Lian), has been used by clinicians to treat bacterial diarrhea. Zhang et al. found that berberine can alter the functions and metabolites of the gut microbiota. And Zhao et al. used berberine to treat a mouse model with acute graft-versus-host disease (aGVHD); their results suggest that the berberine could remodel gut microbiota and prevent colonic barrier impairment (Zhao et al., 2022). Other scholars also found that berberine increased the abundance of beneficial bacteria that can produce SCFAs, and the SCFAs can directly alleviate the accumulation of uric acid (Shan et al., 2022). Song et al. demonstrated that sinomenine can inhibit the inflammatory response by modulating gut microbiota and restoring the intestinal barrier *via* the aryl hydrocarbon receptor/Nrf2-dependent pathway (Song et al., 2021).

**Table 4:** The alkaloid from TCM regulated gut microbiome.

Bioactive ingredients	Detection method	Gut microbiota	Metabolites	mechanism	Ref.
Alkaloids from *Sophora alopecuroides* L.	16S rRNA gene sequencing.	Lactobacillus↑, Oscillospira↓, Desulfovibrio↓.	Fatty acid biosynthesis, lipoic acid metabolism, vitamin B6 metabolism, and so on.	Gut microbiota directly participates in the metabolic process of the host.	(Zhang et al., 2021b)
Berberine	16S rRNA gene sequencing.	Anaerostipes↑, Eubacterium↑, Lachnoclostridium↑, Eisenbergiella↑.	acetate↑, propionate↑, total SCFA↑, valerate ↓, isobutyrate↓.	Altered the functions and metabolites of the gut microbiota.	(Zhang et al., 2021a)
Berberine	16S rRNA gene sequencing.	Adlercreutzia↑, Dorea↑, Sutterella↑, Plesiomonas↑, Lactobacillus↑.	Claudin-1↑, Claudin-2↑, Occludin↑, ZO1↑, LBP↓.	Remodelled gut microbiota and prevented the colonic barrier impairment	(Zhao et al., 2022)
Berberine	16S rRNA gene sequencing.	Coprococcus↑, Bacteroides↑, Akkermansia↑, Prevotella↑.	SCFAs↑.	Beneficial bacteria produced SCFAs, SCFAs alleviated accumulation of uric acid.	(Shan et al., 2022)
Sinomenine	16S rDNA gene sequencing.	Bacteroidetes↑, Proteobacteria↓, Prevotellaceae UCG-001↑, Escherichia-Shigella↓.	Occludin↑, ZO1↑, Claudin-1↑, serum LPS↓, CYP1A1↑, Ah R ↑, NQO-1 ↑, HO-1↑.	Modulated gut microbiota and restored intestinal barrier via aryl hydrocarbon receptor/ Nrf2-dependent pathway.	(Song et al., 2021)

↑ increase. ↓ decrease.

**Table 5:** Chinese herbal compound.

Bioactive ingredients	Detection method	Gut microbiota	Metabolites	mechanism	Ref.
*Astragalus membranaceus* and *Salvia miltiorrhiza* (AS)	16S rDNA gene sequencing.	Lactobacillus↑, Akkermansia↑, Firmicum to Bacteroides (F/B) ↓.	Butyric acid↑, lactic acid↑, ZO-1↑.	Restored the intestinal barrier and flora structure, increased the butyric acid and lactic acid.	(Han et al., 2021)
FuZhengHuaYuJiangZhuTongLuoFang prescription (FZHY)	16S rDNA gene sequencing.	g_Monoglobus↓, g_Papillibacter↓, g_Eubacterium_nodatum↓, g_ Family_XIII_AD3011↓.	ZO-1↑, occludin↑, claudin-1↑.	Reduced the colonic epithelial barrier damages.	(Chen et al., 2022b)
FuFang Zhenshu TiaoZhi(FTZ)	16S rDNA gene sequencing.	Bacteroidetes↓, Proteobacteria↓, Firmicutes↑, F/B ratio↑, Prevotellaceae_UCG-001↑.	TNF-α↓, IL-6↓.	Modulated the balance of intestinal microecology, restored inflammation.	(Luo et al., 2020)
Painong-San extract (PNS)	16S rRNA gene sequencing.	Romboutsia↑, Lactobacillus↑, Bifidobacterium↑, Akkermansia↑, Oscillospiraceae↓, Helicobacter↓.	claudin-1↑, occludin↑, ZO-1↑.	Regulated of gut microbi ota, improved intestinal barrier function.	(Wang et al., 2022a)
Xuanbai Chengqi decoction (XBCQ)	16S rRNA gene sequencing.	Gordonibacter↑, Akkermansia↑, Streptococcus↓, Marvinbryantia↓.	Rectifyed the Th17/ Treg imbalance, TNF-α↓, IL-1β↓, MMP-9↓.	Reshaped the gut microbiota and improved the Th17/Treg balance.	(Wang et al., 2021b)

↑ increase. ↓ decrease.

### 3.2 Chinese herbal compound

Traditional Chinese medicine compound usually consists of two or more medicinal flavors, has relatively prescriptive processing methods and use methods, and is designed for relatively certain diseases and syndromes. At present, many compounds have been applied to the treatment of gut microbiota (**Table 5**). For example, the combination of *Astragalus* membranaceus and *Salvia* miltiorrhiza (AS) is an effective prescription that is widely used to treat chronic kidney disease (CKD) clinically in traditional Chinese medicine. AS could alleviate renal fibrosis and metabolism through the “gut-kidney axis”, Han et al. found that AS restored the intestinal barrier and flora structure and increased butyric acid and lactic acid to exert the above effects (Han et al., 2021). Zhao et al. also found that the Chinese herb FuZhengHuaYuJiangZhuTongLuoFang prescription (FZHY) can effectively treat CKD; the pathways included the regulation of gut microbiota (Chen et al., 2022b). Other researchers found that Chinese herbs have an anti-aging effect. Luo et al. revealed that FuFang Zhenshu TiaoZhi (FTZ) can moderately correct the aging process, which may be related to modulating the balance of intestinal microecology and restoring inflammation (Luo et al., 2020). Painong Powder was developed by Zhongjing Zhang in the Han Dynasty and has been widely used to treat ulcerative colitis (UC) in China for thousands of years. The experimental research confirmed that Painong-San (PNS) extract alleviates colitis in mice by modulating gut microbiota and restoring intestinal barrier function (Wang K. et al., 2022). Xuanbai Chengqi decoction (XBCQ) is a representative traditional Chinese medicine prescription used by Wu Jutong in the Qing Dynasty. It has been widely used to treat a variety of common respiratory diseases in China. There are also scholars studying his role in the regulation of intestinal flora. Wang et al. demonstrated that XBCQ could alleviate chronic obstructive pulmonary disease (COPD) exacerbations by reshaping the gut microbiota and improving the Th17/Treg balance (Wang et al., 2021b). And the studies have shown that Yangyin Qingfei decoction can effectively improve the skin damage of lung cancer patients after radiotherapy, help the growth and propagation of beneficial bacteria in the intestinal tract of lung cancer patients, and regulate the structural balance of intestinal flora (Pan and Zhang 2023).

## 4 Effection of TCMs on prevention and treatment of NSCLC

### 4.1 Active components of TCM

Ganoderma lucidum polysaccharides (GLP) were derived from *Ganoderma lucidum* (lingzhi in Chinese). Gynostemma pentaphyllum saponins (GpS) are derived from *Gynostemma pentaphyllum.* Both are valuable traditional Chinese medicines. Khan et al. provide strong evidence that the cancer-preventive and therapeutic functions of GpS and GLP are through the dynamic modulation of GM and host immune responses. The specific performance is an improved inflamed gut barrier and promoted short-chain fatty acids (SCFAs) producing bacteria (Khan et al., 2019). Saponins, a novel type of plant-derived secondary metabolites, modulate gut microbiota composition and exhibit anti-metastasis activities in multiple tumors, including lung adenocarcinoma, alcohol-induced liver disease, and colorectal cancer (Khan et al., 2019; Chen et al., 2020a; Zhou et al., 2021). Ginsenoside Rh2 (G-Rh2), a major bioactive ingredient in ginseng, suggested the therapeutic effects of G-Rh2 on lung cancer. G-Rh2 regulated the phenotype of macrophages and affected the migration of non-small cell lung cancer (NSCLC) cells (Li et al., 2018). On the other hand, the gastrointestinal microbiome plays an important role in drug metabolism. Ginsenoside is difficult for the molecules to directly exert the pharmacological effects, and the gut flora and its metabolites can improve this process (Yang et al., 2020). A triterpenoid saponin glycoside found in licorice roots is called glycyrrhizic acid (GA). Wu et al. discovered that glycyrrhizin prevented PDX mice from developing lung tumors (Wu et al., 2018). And high amount of High Mobility Group Box 1 (HMGB1) facilitated lung cancer cell invasion and migration, which glycyrrhizin reduced. Here, Qiu et al. found that GA regulates GM to inhibit the establishment of pre-metastatic niches and metastasis that are promoted by HFD by reducing M1-like colonic macrophages *via* LPS/HMGB1/NF-κB signaling. The regulation of intestinal flora is mainly reflected in decreasing the *Clostridiales* order and *Desulfovibrio* genus, and reducing the ratio between *Firmicutes* and *Bacteroidetes* (Qiu et al., 2019). A polysaccharide obtained from Spirulina (PSP) decreased the tumor volume and weight of the lung cancer-bearing mice through regulated arachidonic acid metabolism and the balance of gut microbiota. And the PSP increased the abundance of *Lactobacillus*, *Allobaculum*, *Alloprevotella*, and *Olsenella* and decreased *Bacteroides* and *Acinetobacter* (Lu et al., 2022).

### 4.2 Compound formulation of TCM

Bovis calculus (Bos taurus domesticus Gmelin), Olibanum (Boswcllia bhaurdajiana Birdw., Boszvellia carterii Birdw.), Myrrha, and Moschus are the four herbs that make up the Xihuang pill (XHW), a Chinese medicine formula that has been approved (state medical permit number Z11020073) (Commiphora molmol Engl., Commiphora myrrha Engl.) (Zhang et al., 2021b). It has been widely used in the treatment of a variety of malignant tumors in clinics in China, including breast cancer, lung cancer, gastric cancer, and other malignant tumors (Wang et al., 2020a; Wang et al., 2021a; Ge et al., 2022). In mice with Lewis lung cancer, Cao et al. investigated the anti-lung cancer impact of XHW coupled with anlotinib (LLC). They clarified the regulatory features of XHW in enhancing anlotinib’s anti-lung cancer impact using GM and transcriptomics. The outcomes demonstrated that LLC-bearing mice receiving a combination therapy of XHW and anlotinib effectively suppressed tumor growth. Additionally, XHW’s impact on the control of gut microbiota was significant, as revealed by 16s rRNA sequencing research. The percentage of the helpful bacteria *Bacteroides* and *g_norank_f_ Muribaculaceae* increased as a result (Cao et al., 2022). BuFeiXiaoJiYin (BFXJY) is a traditional Chinese medicine (TCM) compound that has been shown to have good effects in the treatment of lung cancer by ameliorating the NLRP3 inflammation response and regulating gut microbiota. Jiang et al. found that BFXJY reduced the relative abundance of *Firmicutes* and *Verrucomicrobia*, increased the relative abundance of *Bacteroidetes* and *Epsilonbacteraeota*, and enhanced the relative abundance of *Deferribacteres* (Jiang et al., 2022). A Chinese clinical study suggests that Huayu Kangai decoction enema is effective in the treatment of lung cancer and can improve the level of lactic acid bacteria and bifidobacteria (Jia et al., 2023).

In sum, many of the herbal medicines could modulate the relationship between the host and the gut microbiota (GM) to exert their beneficial properties on the host. However, most current studies are mainly one-way studies, which mainly observe the changes in flora and metabolites after the treatment of diseases with traditional Chinese medicine. There will be some problems in this process. For example, Chinese herbs themselves will affect intestinal flora, and intestinal flora is needed for the metabolism and absorption of Chinese herbs. Changes in flora caused by these life processes may not play a major role in the treatment of diseases. In addition, the metabolic components of traditional Chinese medicine are diverse, and some metabolites may be repeated with those of the bacterial community. Therefore, there are many problems in the study of the treatment of lung cancer by regulating intestinal flora with traditional Chinese medicine, and researchers could try their best to improve the experimental scheme and reduce the above problems.

## 5 Conclusion

There are objective differences between lung cancer patients and healthy people in intestinal flora, and more and more studies are gradually proving that by regulating intestinal flora, increasing probiotics, and reducing harmful bacteria content, the cancer inhibitory signaling pathway can be more activated, anti-tumor immunity can be enhanced, thus inducing apoptosis of cancer cells or preventing recurrence and metastasis, and it can play a synergistic role with tumor therapeutic drugs. This paper summarizes and analyzes the regulation of intestinal flora by active ingredients of TCM and Chinese herbal compounds and their intervention effects. Various microbial-related therapeutic means represented by fecal bacteria transplantation and Chinese medicine intervention have been widely used in the fields of diabetes, kidney disease, liver disease, inflammatory bowel disease, and are expected to be popularized in the treatment of cancer, psychiatric diseases, cardiovascular diseases, and so on. Which is conducive to providing new strategies and ideas for clinical prevention and treatment of NSCLC. At present, studies show that TCM regulates intestinal flora to repair the intestinal mucosal barrier and treats NSCLC mainly in two aspects: by affecting immune cells to restore normal immune function of the intestinal mucosa and by improving the sensitivity of antitumor drugs by regulating intestinal flora itself or metabolites. On the other hand, some TCM ingredients cannot be directly absorbed into the blood to play a role in the treatment of diseases, and intestinal flora can achieve drug transformation in this process. With the advantages of multi-target, all-directional, multi-component, and light side effects, TCM intervention in NSCLC has been recognized as a broad prospect. However, the composition of traditional Chinese medicine is complex, and its mechanism of action has not been fully elucidated. In addition, there are a few studies on the treatment of NSCLC through the regulation of intestinal flora by traditional Chinese medicine. Therefore, a large number of basic and clinical studies are needed in the TCM treatment of NSCLC.

## Author contributions

XW and LH collected the articles, MC and JL made the figures. JX guided XW and LH in collecting articles and making figures. MW prerevised the manuscript. XW and LH wrote the manuscript and made the tables. XW and LH have the same contribution to the article. All authors contributed to the article and approved the submitted version.
